# Identifying misconduct-committing officer crews in the Chicago police department

**DOI:** 10.1371/journal.pone.0267217

**Published:** 2022-05-04

**Authors:** Akshay Jain, Rajiv Sinclair, Andrew V. Papachristos

**Affiliations:** 1 Northwestern Neighborhood & Network Initiative, Chicago, Illinois, United States of America; 2 The Invisible Institute, Chicago, Illinois, United States of America; 3 Public Data Works, Greensboro, North Carolina, United States of America; 4 Department of Sociology, Northwestern University, Evanston, Illinois, United States of America; 5 Institute for Policy Research, Northwestern University, Evanston, Illinois, United States of America; London School of Economics, UNITED KINGDOM

## Abstract

Explanations for police misconduct often center on a narrow notion of “problem officers,” the proverbial “bad apples.” Such an individualistic approach not only ignores the larger systemic problems of policing but also takes for granted the group-based nature of police work. Nearly all of police work is group-based and officers’ formal and informal networks can impact behavior, including misconduct. In extreme cases, groups of officers (what we refer to as, “crews”) have even been observed to coordinate their abusive and even criminal behaviors. This study adopts a social network and machine learning approach to empirically investigate the presence and impact of officer crews engaging in alleged misconduct in a major U.S. city: Chicago, IL. Using data on Chicago police officers between 1971 and 2018, we identify potential crews and analyze their impact on alleged misconduct and violence. Results detected approximately 160 possible crews, comprised of less than 4% of all Chicago police officers. Officers in these crews were involved in an outsized amount of alleged and actual misconduct, accounting for approximately 25% of all use of force complaints, city payouts for civil and criminal litigations, and police-involved shootings. The detected crews also contributed to racial disparities in arrests and civilian complaints, generating nearly 18% of all complaints filed by Black Chicagoans and 14% of complaints filed by Hispanic Chicagoans.

## Introduction

Police misconduct, abuse, and violence takes a heavy toll on civilians, especially Black and Latino communities. Although figures on police-involved killings are not without debate, recent estimates suggest that from 2012 to 2018 police in the U.S. killed approximately 2.8 men per day, with Black men facing mortality risks from police homicide that are three-times higher than those faced by white men [1]. The impact of police misconduct and abuse extends far beyond loss of life and can impact physical and mental health [2, 3]. Furthermore, police shootings represent only the most extreme and visible form of police abuse. For every shooting reported in the media, there are thousands of instances of non-lethal use of force, verbal abuse, demeaning interactions, and other problematic police behaviors. For example, between 2004 and 2014 police in Chicago fired their weapons at citizens approximately 694 times, deployed their tasers 3,646 times, and were involved in more than 60,000 other forms of non-lethal interactions that required some “tactical response” [4]. Although some of these instances may not constitute “misconduct” under legal statutes, each has a consequence for civilians subjected to force. In addition, such figures likely fail to capture the interactions between civilians and police–ranging from demoralizing to traumatizing–that generate feelings of mistrust, cynicism, and estrangement further undermining the relationship between the community and police that is crucial for public safety [5–7].

As the country wrestles with the racialized history and systemic problems of policing, the development of policy responses is often guided by a narrow focus on the notion of “problem officers,” the proverbial “bad apples.” While some evidence supports the proposition that a small portion of officers generate a disproportionate number of citizen complaints and use of force reports [8, 9], such an individualistic approach ignores not only the larger systemic problems of policing but also the group-based nature of police work. Officers do not work in a vacuum. Much of police work is group-based through assignments to geopolitical districts, units, precincts, work schedules, or simply being partnered with other officers. Recent research has begun documenting the group nature of police misconduct and violence. Like deviance more generally, much of police misconduct is a group behavior related to the informal and formal networks among officers [10–12]. Police misconduct, including the use of force [13] and shooting at civilians [14] is correlated with the structure of such networks and officers’ behavior appears to be influenced by the officers with whom they work [11, 15].

The misconduct networks documented in this emerging research by and large refer to informal networks created by police on the job or through work assignments. But in extreme cases, groups of officers have even been observed to coordinate their abusive and even criminal behaviors. The Los Angeles Rampart scandal, for instance, involved over 70 police officers in the Los Angeles Police Department (LAPD) who were involved in assaults, drug crimes, fabricating evidence, perjury, and, allegedly, murder [16]. During the same time period, four officers in Oakland known as the “Riders” were accused of actions ranging from filing false reports to assault, kidnapping, and severely beating civilians with their fists, feet, pepper spray, and metal clubs [17]. The groups of officers involved in both Rampart and Riders cases were far more than “bad apples” and, instead, coordinated their actions for the furtherance of their criminal activities as well as to avoid detection. The Rampart crew developed its own group symbols, oaths, and language, contributing to the overall sentiment that the involved officers acted more like a gang than a police unit.

If single officers can have a disproportionate impact on police violence, it stands to reason that officer “crews,” as we will refer to them in this study, are likely associated with even higher levels of violence and misconduct. For instance, the officers in the Rampart scandal have been linked to more than 100 overturned criminal cases and more than 140 civil lawsuits that resulted in more than $125,000,000 in settlements. Yet virtually nothing is known about how many such crews exist within departments, how much harm they are responsible for, and how to detect them. One reason for the elusiveness of these crews—besides their active role at concealing their behavior—is the difficulty of group identification. If one only sees individual “problem officers,” the rest of a network might seem invisible. Furthermore, it is difficult to ascertain characteristics of known crews since most of our knowledge of such groups stems not from individual complaints by civilians but, instead, from whistleblowers and subsequent internal and external investigations.

This study adopts a social network and machine learning approach to empirically investigate the presence and impact of officer crews engaging in misconduct in a major U.S. city: Chicago, IL. Using public data on Chicago police officers between 1971 and 2018, we seek to identify potential crews and analyze their impact on police misconduct and violence. We begin by creating a social network using complaint and use of force data and identifying communities of officers within the network using known crews as a performance metric. Using properties of existing crews known through litigation and investigation, we then develop a “crew index” to help identify potential crews within the larger departmental network. We conclude our analysis by evaluating the impact of the communities detected through our approach on levels of misconduct and police behavior in the city. While we cannot and do not propose this analysis to be considered predictive, it serves to be a useful exploratory tool and launching-off point for future analysis and investigations.

## Background

We use the term *police misconduct* broadly to refer to “illegal or unethical actions or the violation of individuals’ constitutional rights by police officers in the conduct of their duties” [18]. The most pervasive explanation for police misconduct is that of the “problem officer,” i.e., that misconduct is the result of some error in judgement or else some character flaw, predisposition, bias, or lack of self-control on the part of problem officers [19–21]. The policy implication of such an individualistic framework is straight-forward: find (and do something about) the problem officers. Several findings emerge from prior research relating to individual officer traits and characteristics. Female officers are less likely to engage in misconduct, are less likely to use weapons or cause injury during use of force situations, and receive fewer complaints as compared to their male peers [22, 23]. Younger and less experienced officers receive more complaints—including use of force complaints—than older and more experienced officers [21]. Findings on education are mixed, though mounting evidence suggests that some college education is associated with lower levels of misconduct [24, 25]. Misconduct also appears to be associated with anti-social behavior of officers and personality factors such as low self-control and an “authoritarian personality” [26, 27]. Findings on the association between the race and ethnicity of an officer and misconduct are somewhat mixed [28]. Some studies suggest that Black officers receive fewer complaints on average than their white counterparts [29, 30], other studies have found higher rates of complaints among non-white officers [31], and some research has found no difference in shootings or use of force between white and non-white officers [21, 32]. Moreover, some of these individual-level findings are conditioned on contextual factors that can change the interactions police have with citizens, especially high-conflict interactions [33, 34].

The problem officer approach is frequently criticized for its conception of officers as atomized individuals divorced from the history of policing as an institution and the impact of larger police organizational culture. Problematic or not, officers are part of a larger hierarchical organization with its own culture, practices, policies, and history. While officers enact considerable discretion in individual encounters and interactions, the structure of police organizations shape some of the most basic police duties and interactions—especially where officers work, the type of policing they will do, who they police, and with whom they police. Such policies and structures underscore a fundamental dimension of policing: policing is a *group* phenomenon in which officers are influenced by formal and informal networks and structures; history and organizational culture play a key role in creating and maintaining these sorts of networks and groups. Individual officers work in organizations, are assigned to districts, shifts, teams, units, and work particular assignments and beats. These different assignments are staffed from pools of officers who, as humans do, form ties and social bonds between co-workers. Officers work with each other on the job. They attend trainings and roll call together. They go through the academy and work cases together. And, of course, many form off-the-job friendships and personal relationships.

These sorts of networks and relationships, both formal and informal, matter. Formal police organizational networks—supervisors, co-workers, instructors, and so on—provide one of the first and primary sources of the learning of police norms, rules, and behaviors. But officers learn social norms informally from their fellow officers as well. Ethnographic research underscores that one’s fellow officers provide information about work culture, behavioral norms, and provide positive (or negative) reinforcement of behaviors. For example, Savitz demonstrates how new police recruits evolve from the rules of behavior taught at the police academy to those modeled by officers already on the job: within a short period of time, new officers’ attitudes became more permissive to match the views of their departmental colleagues [35]. Likewise, a network study of police found that informal friendships developed during training can have a more powerful impact on attitudes surrounding race and diversity than formal training protocols [36].

The formation and importance of these sorts of networks is by no means unique to policing. The field of network science continually demonstrates ways in which the social connections among individuals impact a range of behaviors from the votes we cast and the things we buy to our health, happiness, and well-being [37–39]. Social networks themselves, by which we mean a set of social connections and relationships among a set of actors, are created and maintained by the interactions among individuals. At the same time, networks are also shaped by extra-individual forces. In this way, networks provide a meso-level explanation of human behavior that falls somewhere between the bottom-up theories of atomized individuals and the top-down theories of organizational culture. Network levels of explanation are also central to theories of crime and deviance [40, 41]. From street corner violence [42] and school yard bullying [43] to corruption [44] and human trafficking [45], network science continually demonstrates that individual deviant behavior is impacted by the networks in which individuals live, work, and act. Deviance is learned behavior and such learning occurs within peer, work, school, and neighborhood networks [46].

Studies have increasingly paid attention to the importance of networks on police behavior [10, 11, 13, 15]. Three key findings have emerged from this research. *First*, like deviance more generally, police misconduct appears to be a group phenomenon. In a recent Chicago study, more than 50 percent of all civilian complaints filed against police listed more than 1 officer with approximately 15 percent of complaints listing more than 2 officers [10]. Pairings of officers in the misconduct network are decidedly non-random and alleged misconduct is more likely to occur between some subsets of officers from a larger pool of possible coworkers, partners, and associates [10].

*Second*, misconduct does not appear to be evenly distributed within departments. Studies using data from Chicago found that while the most common number of complaints for an officer was 1, there was a highly skewed distribution with max of 162 complaints and a standard deviation of 11.2; furthermore, approximately 17% of all officers were responsible for 50% of all complaints [8, 10]. Such problem officers within networks are far from isolated individuals and might, in fact, have an outsized impact on misconduct. Zhao and Papachristos [14] find that high-complaint generating officers tend to occupy structural positions as “brokers” *between* other officers in the network suggesting that some of these problem officers might, in fact, be the glue that holds misconduct networks together, either by their continued presence in certain parts of the networks or as police leaders shuffle problem officers around the organization. What’s more, officers occupying brokerage positions are three-times more likely to shoot at a civilian, even when that civilian posed no threat to the officer or bystanders [14].

*Third*, there is some evidence of peer influence of misconduct and use of force within officer networks where an officer is likely to adopt similar patterns of use of force as their partners and peers. For example, Field Training Officers (FTOs) responsible for the “on the job training” of new recruits exert tremendous influence on behavior; one study found that roughly one quarter of the variation in new police officers’ allegations of misconduct were attributable to FTOs [47]. Even informal relationships can influence use of force. A recent Chicago study by Ouellette et al [13] found that, over time, as more of an individual officer’s peers engage in use of force behaviors, so too will that focal officer engage in use of force patterns similar to their peers. While these studies are not direct evidence of contagion in a causal sense, they are consistent with social learning theories of police misconduct [15] and suggestive of peer effects.

These network studies show that the idea that misconduct is mainly the result of problem officers is overly simplistic. Not only do these problem officers exert tremendous influence on use of force, they are also key factors that influence larger misconduct patterns, perhaps even being linked to the spread of such behaviors within police departments. More simply put, the idea that misconduct emerges out of atomized choices of disconnected individuals ignores the influence of larger structures on officer behavior, especially the key role of peers, working groups, and networks. A network approach to police violence offers a unique perspective that can capture both individual variation in behaviors as well as the larger structures which may impact individual behavior, including formal structures (such as assignments) and informal relationships (such as those formed through behavior or friendships).

### Looking for crews: Social network analysis and community detection

Since its inception, one of network science’s foundational analytic tasks has been the identification and analysis of subgrouping within larger networks [47]. Dozens of different analytic techniques and algorithms exist for detecting subgroupings, cliques, or clusters within larger networks, all with roughly the same goal of identifying “meaningful” subgrouping of individuals.

Identifying criminal and deviant cliques is especially difficult since such networks go to great lengths to avoid detection and conceal their behaviors [41, 44]. The same is likely true of police crews. To date, most of the information about networks of bad cops comes from whistleblowers or completed investigations known to authorities. Often such cases begin and end with the few crew members whose behavior became known to investigators or journalists. Such a focus on singular cases limits our knowledge of the extent of such wrong-doing while simultaneously reinforcing the “problem officer” narrative. Quite simply: by focusing on “known cases” we likely underestimate the scale or impact of such crews on police misconduct.

Our study is guided by past research attempting to identify communities within larger criminal networks. This area of research often begins with known cases used to detect certain network or structural signatures that then get expanded to look for subroutines within a broader network. For example, Calderoni et al. used community detection algorithms to look for subgroups or “locales” within the Ndrangheta mafia organization in Italy [48]. Based on data of meetings between participants obtained from the law enforcement operation “Operazione Infinito,” the authors constructed a network and used the modularity-based Louvain algorithm to partition the network into communities. The study then uses several structural characteristics of the network to identify likely communities for unlabeled nodes as well as identify the probable “boss” of each community.

Similarly, Bahulkar et al. employ community detection algorithms and link-prediction algorithms to account for deliberately hidden connections in in two criminal networks, the Ndrangheta mafia and a Canadian drug trafficking network [49]. Bahulkar et al. found that while link prediction can increase the power of community detection, they do not offer substantial improvements in cases when the deliberately hidden edges were important for community formation. The study also reinforced the idea that while community detection is often a useful tool in network science, the existence of prior knowledge, which could include an individual’s role in a hypothetical community, can greatly improve the results.

While our study extends this prior work, the nature of police organizations requires some additional considerations. Officers *are* part of the larger police department and specific units or assignments, but need *not* be part of a criminal crew within the larger police organization—i.e., some officers might be linked to crews through assignment or chance as opposed to active engagement or association. In some known cases of police misconduct, individual officers may appear to belong to a crew within the larger network for the sole reason of only working with a particular subset of officers based on assignment but might not be part of any misconduct or illegal behavior. This presents an empirical challenge as we must then differentiate detected communities that are simply a byproduct of the larger organizational structure of policing from subgroups of officers actually engaging in coordinated misconduct or criminal behavior.

The main objective of the present study is to detect the presence of smaller intentional cop crews within a larger police organization to (1) determine the frequency of such crews and (2) the extent such crews contribute to overall patterns of police misconduct. We start by looking at all patterns of individual and group misconduct but differentiate “crews” based on four central criteria which we derive from known cases of misconduct discussed below:

1. *Frequency*: the officers display high levels of instances of alleged co- misconduct more generally;
2. *Exclusivity*: the officers are involved in recurrent co-misconduct *within* the group, as opposed to co-misconduct with external officers;
3. *Severity*: officers in the same crew are engaged in similar types of alleged misconduct activities as each other (homophily in misconduct patterns); and
4. *Cohesion*: the group exhibits well-defined membership.

Regardless of individual attributes, officers are more likely to receive complaints when they are assigned to geographic communities experiencing higher levels of crime in large part since officers patrolling or assigned to those areas are more likely to have greater contact (in general) with residents and, thus, greater opportunities for misconduct to occur. While high-crime areas may have more complaints leading to groups with higher rates of joint misconduct, such a fact does not mean that the group is not a crew. In fact, the known crews used here appear at a disproportionately high rate in high-crime areas. As such, the confounding effect of geography with frequency is less problematic in our present study, but future research should consider other ways in which the geographic distribution of both crime and policing might impact police practices and behavior, including the formation of crews such as those studied here.

In the present study we use a unique set of data on all instances of complaints over an extended period of time for a city with a long history of police misconduct, including several known high-profile cases of officer crews: Chicago, IL.

### Police misconduct in Chicago

The Chicago Police Department (CPD) is the nation’s second largest police department with more than 13,000 sworn officers and civilian personnel, and an annual operating budget of more than 1.7 billion dollars [50]. CPD’s history of misconduct, abuse, and corruption follows the department throughout its history, with defining moments of CPD involvement in labor strikes of the late 1800s and early 1900s, the 1919 Race Riot, and a legacy of systematic corruption throughout the Prohibition Era [51, 52]. CPD’s history of abuse continued in the 1960s with the world-wide coverage of the 1968 Democratic National Convention, their role in the assassination of Fred Hampton, and the activities of the Gang Intelligence Unit whose involvement in Cointelpro and unconstitutional surveillance of Black and Latino gangs and organizations. The high-levels of misconduct coupled with a lack of effective oversight and accountability continues to this day. In January 2017, the United States Department of Justice released an expansive report that documented repeated and frequent instances of excessive use of force, misconduct, and derogatory language and behaviors towards civilians, especially minority civilians [53]. The 2017 report further revealed that investigations into acts of alleged misconduct were often incomplete and unfair, in addition to being understaffed and impeded by city policies. The Justice Department claimed that investigations did not effectively deter future misconduct, nor did CPD or the city take sufficient steps to prevent officers from concealing acts of misconduct. The report noted that of the over 30,000 annual complaints against CPD officers, less than 2% are sustained, and the city does not investigate a majority of the cases they are legally obligated to investigate.

Without a doubt, there are plenty of “problem officers” throughout CPD’s 185 year history. Our objective is not to point out specific problem officers but, instead, those crews of cops who actively form groups for the purpose of furthering or concealing their misconduct or illegal behavior. Given the group and partner-oriented nature of policing in Chicago, one of our foundational analytic tasks is to differentiate group misconduct that is a product of the organizational structure of police (e.g., two officers who receive a complaint merely as a function of working together) from those instances of more intentional crews. To this end, we develop and assess our approach based on three known cases of cop crews: The Watts Crew, The Skullcap Crew, and The Austin Seven.

#### Watts crew

For nearly twenty years, Ronald Watts, Kallat Mohammed, and a group of more than 10 other officers were involved in high levels of misconduct and criminal activity in and around the Ida B. Wells public housing complex on the South Side of Chicago [54]. Officers in this “Watts crew” were an integral part of the drug trade in the communities they policed. They extorted a tax from drug dealers for police protection, targeted the competition of those dealers under their protection, and would seize the drugs for their own distribution [54]. Many residents have come forward to recount being framed on drug charges by members of the Watts crew when they refused to participate in their criminal ends. Since 2012, more than 80 individuals have had their convictions overturned due to the involvement of these officers, yet the true impact of the Watts crew could likely be far greater [55]. Supervisors repeatedly dismissed allegations against the involved officers and intimidated any officer who sought to report wrongdoing. In 2012, Watts and Mohammed were apprehended and later convicted after they attempted to steal money from an FBI undercover agent disguised as a drug courier. Other officers in the crew have thus far not been criminally charged.

#### Skullcap crew

In the early 2000s, an investigative journalist detailed the experiences of a public housing resident who was repeatedly physically, verbally, and sexually assaulted over an extended period of time by a group of five CPD officers known to the residents as the “Skullcap Crew” [56]. These five officers are alleged to have committed high levels of misconduct and brutality against other public housing residents and were involved in many reported instances of excessive and unwarranted force, sexual abuse and harassment including strip searches, planting drugs, theft, and false arrest [57]. Altogether, officers who were part of the Skullcap crew received 138 allegations of misconduct, 39 of which included more than one officer from the crew. Few of these complaints were sustained or acted upon, and four of the five officers remain on the force and have even earned departmental commendations.

#### Austin-Seven

The Austin-Seven was a group of seven police officers, named for the Austin neighborhood in their assigned police district, who in the early 1990s were involved in cases of robbery, extortion, and drug dealing [58]. Similar to the officers in the Watts crew, the Austin-Seven allegedly collected money from drug dealers in exchange for protection and used their firearms to assist local drug dealers and gang members in robberies and other violent crimes. In 1995, the CPD internal affairs department received a tip about the officers and passed the information along to the FBI, which then conducted a successful undercover operation posing as drug dealers. The seven officers were charged with 21 separate counts of conspiracy to commit robbery and extortion, and illegal use of firearms. Locker searches of several of the officers revealed drugs as well as evidence that a particular officer was affiliated with a street gang.

### Data

This study relies on several unique sources of data collected through FOIA and litigation requests by The Invisible Institute, a non-profit journalism company on the South Side of Chicago. The present analysis combines data from **seven** unique sources relating to complaints of police misconduct, personnel data, officer assignments, city settlement data, arrest data, use of force reports, and officers in known crews. This study focuses on the time period from 1971 to 2018, though some of the listed datasets only cover a subset of this time period.

### Complaint data

We use data on allegations of misconduct filed against officers of the Chicago Police Department to estimate police misconduct activity. From 1971 to 2018 approximately 131,414 allegations of misconduct were filed against 23,444 individual officers. Complaints were filed by both civilians and fellow officers. During the observation period, the mean number of allegations per officer is 10.2 (min = 1, max = 162, SD = 11.1); the mode of complaints per officer was 1. Just less than half of all allegations (46.2%) list more than one officer, with an average of 1.8 officers listed on each allegation (min = 1, max = 192, standard deviation = 1.7). The complainant’s race and gender are listed for 32,944 of total complaints (25.1%), the vast majority of which occur after 2006 due to data limitations.

An allegation of misconduct does not necessarily mean misconduct has occurred. At the same time, we should not assume that unsustained allegations are necessarily unfounded or in bad faith [59, 60]. Even if an unfounded allegation does not meet the legal criteria for misconduct, it may still constitute “problematic” behavior that negatively affects public trust and confidence in the police [6]. Despite these limitations, some prior research find that complaints do in fact capture instances of problematic police behavior and misconduct.[19] A recent study using these same data finds that despite low levels of substantiation of complaints, a strong relationship exists between civilian complaints and future civil litigation [8].

#### Personnel data

We rely on publicly available information on CPD personnel to ascertain officer gender, race, appointment date, rank, resignation date, and other individual-level information. 61.8% of the 32,939 CPD officers listed in the data are white, 22.6% are Black, and 13.50% are Hispanic. The majority of officers (82.9%) are male and 17.1% are female. Personnel data were successfully linked to 96.9% of the officers listed in the complaint data, including 10,234 officers who did not receive any allegations.

#### Officer assignment data

Data on officer assignments allows us to identify the unit of each officer during their time of appointment at CPD and throughout their career. This dataset provides each officer’s name, unique identifier, the numerical code of the assigned unit, and the corresponding date range. This dataset contains 29,918 unique officers and 216 unique unit codes. Including reassignments to a previous unit, each officer in this dataset served in an average of 3.6 units (min = 1, max = 27, standard deviation = 2.8).

#### Settlements data

We link individual officers to settlement data paid by the City of Chicago in civil cases pertaining to alleged instances of misconduct. This dataset consists of 930 total settlements from 1993 to 2016 (a subset of our total data) and lists 2,350 unique officers. This data contains information regarding the case number, officer, plaintiff, incident date, and settlement amount. The average settlement amount was $244,056 (min = $350, max = $15,000,000, standard deviation = 1,101,618).

#### Arresting officer data

We link misconduct-committing officers to arresting officer data in order to identify the number of arrests made by each officer. The data is limited to arrests made between 2001 and 2018 and consists of 2,507,197 total arrests and includes 17,557 unique officers.

#### Tactical Response Report (TRR) data

To capture instances of reported use of force, we rely on Tactical Response Reports (TRR) which all CPD officers are required to complete after using substantial physical force against a civilian or after a civilian claimed to have been injured by the police officer. This dataset, which covers the period 2004 to 2016, documents 84,729 separate TRR reports for 47,476 separate events and lists 10,799 unique officers. As a TRR is self-filed, it is possible that officers are engaged in unreported uses of force.

#### Known crews officer lists

To obtain a list of the officers who were a part of the four crews listed above, this study located names of the involved officers from investigative journalists at the Invisible Institute and other newspaper reporting, especially *The Chicago Tribune* and *The Chicago Sun-Times*. The sources together revealed 29 total names, corresponding to 17 officers belonging to the Watts crew, seven officers belonging to the Austin-Seven, and five officers belonging to the Skullcap crew. It is important to note that the inclusion of these names is based on a combination of investigations and testimony; there could be more officers who were involved in these crews who were not listed, and conversely, there could be officers who were listed who should not have been included. These public cases—especially those that have already led to legal actions—provide a documented starting point for the existence of crews, and, as such, also serve as a helpful starting point for community detection algorithms.

## Methods

The Northwestern University IRB reviewed this protocol and deemed it "not human research" (IRB protocol STU00214685).

### Network creation

Our analysis proceeds in three stages. The first stage re-creates the co-accusal network of complaints filed against all CPD officers from 1971 to 2018. This network only includes officers who have received more than one complaint, as officers who have received only one complaint are presumably not part of crews. In constructing the network, individual officers are treated as the “nodes” while the “edges” connecting officers are instances of co-accusal on a complaint. Hence, two officers are linked if they have at least one co-complaint.

Edges are weighted by the number of co-accusals and an inverse fraction corresponding to the number of officers listed on each complaint to reflect the strength of a tie, especially when there are fewer officers. This may seem counterintuitive given the focus on communities. However, community detection algorithms perform better when edges are defined by reducing noise and increasing the prevalence of meaningful edges. If, for example, there are twenty officers listed on a complaint, the level of interaction between each of those officers is conceivably lower than if there were just two officers listed on a complaint.

As such, if two officers, represented by ‘k’ and ‘j’ share N complaints, then the edge weight connecting the two nodes is represented by Eq 1.


$$w(k,j)=\sum_{i=0}^{N}(1+{\frac{1}{\mathrm{Number}\;\mathrm{of}\;\mathrm{officers}\;\mathrm{Listed}\;\mathrm{on}\;\mathrm{Complaint}}}_{i})$$ (Eq 1)

All edges of a weight less than two are then removed from the network, followed by the removal of all isolates (nodes with no edges). What remains is a co-accusal network with 11,227 nodes and 26,331 edges. The average degree for a node in this network is 4.69 (min = 1, max = 60, standard deviation = 6.09), meaning that each officer is connected to an average of 4.69 other officers. The average weighted degree is 19.31 (min = 2.02, max = 568.19, standard deviation = 31.79), meaning that each edge has an average weight of 4.12 (see also, Appendix A in S1 File).

Given the nature of police misconduct, certain acts may not be reported and certain ties may be deliberately hidden, which could have a possible impact on the network structure and community detection attempts. While some studies of criminal networks attempt to rectify this problem by using predictive edge algorithms [49], we choose not to add any non-reported complaints to err on the side of caution given the sensitive nature of identifying crews of police officers.

We treat ties in the aggregate, essentially flattening the entire time period, for two reasons. First, the observed ties themselves are proxies for actual misconduct and likely represent only a portion of actual misconduct among officers. As a case in point, all of the existence of known cases we analyze come from whistleblowers not directly misconduct reports themselves. Second, data from earlier in our time window are somewhat sparser and dividing the data temporally would likely entail dropping some of the known examples we use for model calibration. While treating these data as static takes for granted temporal and historical patterns, supplemental analyses that consider parsing the data into discrete time periods found that even partitioning the networks into smaller time frames does not alter our main findings (see S1 File). The objective of the present study is to establish the ability to possibly identify crews not to unpack the temporal conditions that give rise to them; future research requiring additional data and temporal modeling should consider how these crews come to exist or evolve over time.

### Community detection

The second stage of analysis involves *community detection* within the co-accusal network to identify highly connected subsets of nodes within a larger network. Such algorithms are used in a variety of contexts ranging from identifying communities in criminal networks to locating associations between product preferences [61, 62]. The intuition behind most community detection algorithms is to identify subgraphs with nodes that are more connected with other nodes *within* the subgraph than to nodes *outside* the subgraph. The present study employs four different, well-known community detection algorithms to detect clusters of officers within the larger co-accusal network: the Louvain method, K-Clique Percolation, Label Propagation, and the Clauset-Newman-Moore greedy modularity maximization algorithm (see Appendix B in S1 File). We selected the Louvain method at a resolution of 0.006 as it significantly outperformed the other algorithms in terms of detecting known crews. It is important to note that with the Louvain algorithm, each officer is assigned to a community. Since not every officer in the network is truly in a “crew” as we define it, it is necessary to identify which of these communities are in fact crews of the sort of interest here.

### Crew identification

**Fig 1:**
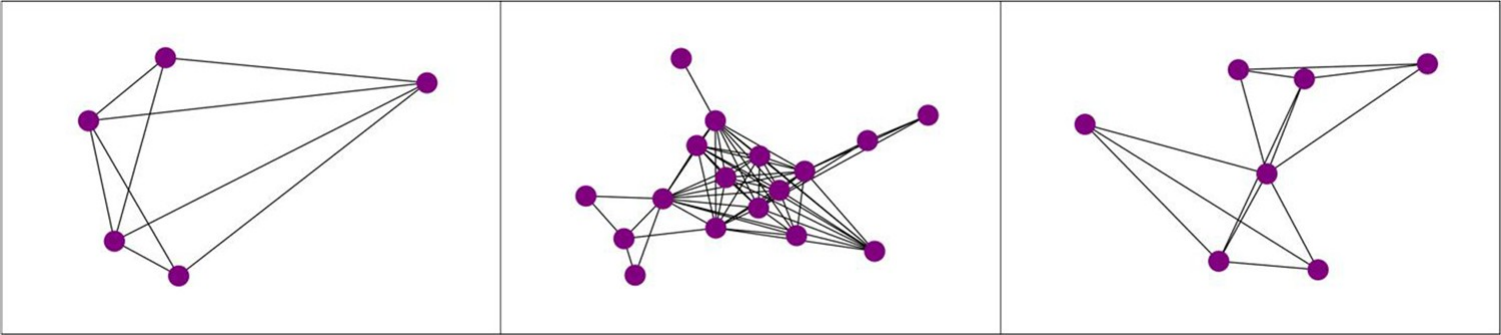
From left to right: Skullcap crew, Watts crew, Austin-Seven crew. Depicted edges are unweighted.

Perhaps the most difficult and novel component of this study is identifying the likelihood that each detected community is an actual crew. Complicating matters is the fact that the structure of crews likely vary. To add in our validation that communities might represent crews, we re-created the co-accusal networks of the known crews to calibrate our detection. Our analysis reveals variation in crews in terms of their edge formation and network structure (see Fig 1). Any attempt to determine crews based solely on structural similarities is therefore limited. Additionally, as a function of the small sample size of crews, it is impossible to generalize based solely from these three examples. In other words, our approach attempts a classification problem without a training set. To account for this problem, we generate a standardized probability distribution based on a linear combination of variables that are directly and positively correlated with the likelihood of a community existing as a crew. This probability does not reflect the probability that the community is a crew, but rather the relative probability when compared to all other communities. A linear combination was chosen over a multiplicative product of the variables to eliminate the interaction effect that those variables would have.

We augment detection using structural properties with four variables mentioned above that are directly correlated to the likelihood that a community is a crew. These variables represent characteristics of known crews and offer metrics for analysis.

#### Frequency

To measure high levels of co-accused misconduct, we use the average weighted degree of a community. An officer’s weighted degree will account for the number of partners as well as the frequency of co-accused misconduct.

#### Exclusivity

To measure internal activity of a crew, we use the total number of within-community complaints (also referred to as “internal complaints”) as well as the number of within-community complaints per member. As noted by Wachs and Kertész [63], a group’s exclusivity, with respect to their network structure, is directly proportional to the likelihood that they exhibit markers of cartel behavior, or crew behavior in this case. Inspired by the z-P analysis used in Guimera and Amaral [64] and Calderoni et al [48], we use two separate variables in order to discount any bias towards larger communities.

#### Severity

Attempts to study known crews as well as public information on crews suggest a correlation between the *types* of complaints received and officers in crews. We use an unsupervised clustering algorithm on officer complaint breakdowns to identify different types of officers with respect to the complaints they receive. To increase the generality of the results, several complaints were grouped together. Table 1 lists the five types of complaint reductions. In addition to including the percentage of each of these five types, we also include the total number of allegations, the percent of received complaints with no other officer listed, the percent of complaints that were sustained, and the percent of complaints that were unfounded or exonerated.

**Table 1:** Typology of complaints used for officer categorization.

Type 1	Type 2	Type 3	Type 4	Type 5
Use of Force	Bribery/Official Corruption	Racial Profiling	Operation/Personnel Violations	Drug/Alcohol Abuse
Illegal Search	Money/Property	Verbal Abuse	Lockup Procedures	Domestic
False Arrest		First Amendment	Supervisory Responsibilities	Conduct Unbecoming (Off-Duty)
Criminal Misconduct			Traffic	
			Medical	

We employ k-means clustering and test a variety of k-values. The optimal k-value of eight was chosen through average silhouette score analysis (see Appendix C in S1 File). This provides each officer with a category label from one to eight. Table 2 shows that known crews feature a disproportionately higher percentage of category four and six officers. As such, category four and six officers are collectively referred to as “flagged officers” and will serve as the metric for measuring officer composition of detected communities. See Appendix D in S1 File for the average values of clustering variables for each officer category.

**Table 2:** Officer category breakdown by known crew. All officers column includes officers who also did not receive misconduct allegations.

Officer Category	All Officers (%)	Watts Crew (%)	Skullcap Crew (%)	Austin Seven Crew (%)
1	3.06	0	0	0
2	0.37	0	0	0
3	5.48	0	0	0
4	23.81	41.18	0	71.43
5	30.55	0	0	0
6	9.42	41.18	100.00	28.57
7	3.37	0	0	0
8	23.94	17.65	0	0

#### Cohesion

Communities show higher cohesion if their existence as a community is validated by another community detection algorithm with a different methodology. As also noted by Wachs and Kertész [63], in addition to their exclusivity, a group’s cohesion is directly proportional to the likelihood that they exhibit markers of cartel, or crew, behavior. We use a label-propagation approach as the verifying algorithm. To obtain a metric for each Louvain community, we take the most frequent label propagation community ID among the members and calculate the Jaccard similarity score. If the Louvain algorithm and label-propagation were to identify the exact same community, the Jaccard similarity score for that community would be 1.0 and would reflect the increased probability that the community was a crew.

Each of the four metrics—frequency, exclusivity, severity, and cohesion—are standardized and then placed into a linear equation with equal coefficients. Equal coefficients provide each variable with the same effect since no other weights can be inferred without additional data. Future studies may seek to better calibrate these weights to correspond to the relative importance each component has on the overall probabilistic effect. Eq 2 represents the raw overall score of each community which is then scaled and standardized between 0 and 1 to act as the crew probability, as seen in Eq 3. Again, the probability indicates relative probability compared to other communities, rather than conclusive probabilistic evidence of crew existence. Given the fact that the index is an equally weighted and standardized linear function of the four components, two communities may obtain the same index score in different ways. For example, one community may have high frequency and exclusivity scores and low severity and cohesion scores, while another community may have high severity and cohesion scores and low frequency and exclusivity scores, presenting them with similar index values. Furthermore, the standardizing effect exemplifies the importance of outlying values. For example, if a community has the highest severity score by a large margin compared to all other communities, the index function will represent that accordingly. The following represent the quantification of the four metrics: frequency (*W*), exclusivity (*T* and *I*), severity (*F*), and cohesion (*J*).

Let,

A=setofallofficersinnetwork

C=setofalllouvaincommunities(eachcommunityisasetofofficers)

$$n_{X}=number\;of\;officers\;in\;louvain\;community\;‘X’$$

flagged=setofallflaggedofficers

$$L_{X}=label\;propagation\;community\;corresponding\;to\;community\;X$$

g(X)=NumberofcomplaintslistingmorethanonememberofcommunityX

And let,

$$W=\left\{\frac{1}{2n_{x}}\sum_{i\in X}\sum_{j\in A}w(i,j):\forall X\in C\right\}$$

T={g(X):∀X∈C}

$$I=\left\{\frac{1}{n_{x}}g(X):\forall X\in C\right\}$$

$$F=\left\{\frac{1}{n_{x}}\sum_{i\in X}1_{flagged}(i):\forall X\in C\right\}$$

$$J=\left\{\frac{\left|X\cap L_{X}\right|}{\left|X\cup L_{X}\right|}:\forall X\in C\right\}$$

Note: ∀*i* = *j*, w(*i*,*j*) = 0

For the sake of notation, let a set followed by a subscript ‘x,’ represent the element of that set corresponding to community x. Then,

$$R(x)=\frac{W_{x}-\mu_{W}\;}{\sigma_{W}}+\frac{T_{x}-\mu_{T}}{\sigma_{T}}+\frac{I_{x}-\mu_{I}}{\sigma_{I}}+\frac{F_{x}-\mu_{F}}{\sigma_{F}}+\frac{J_{x}-\mu_{J}}{\sigma_{J}}$$ (Eq 2)

$${Pr}_{crew}(x)=\frac{R(x)-{argmin}_{x\;\in \;C}(R(x))}{{argmax}_{x\;\in \;C}(R(x))-{argmin}_{x\;\in \;C}(R(x))}$$ (Eq 3)

## Results

### Community detection results

The Louvain algorithm identified 2,334 communities. The mean size of a community is 4.81 (min = 1, max = 23, standard deviation = 3.71). The present analyses includes only communities of a size greater than two to capture groups that might result from more than administrative partnerships and dyadic misconduct: 1,331 communities (a total 9,272 officers) had more than two members.

**Fig 2:**
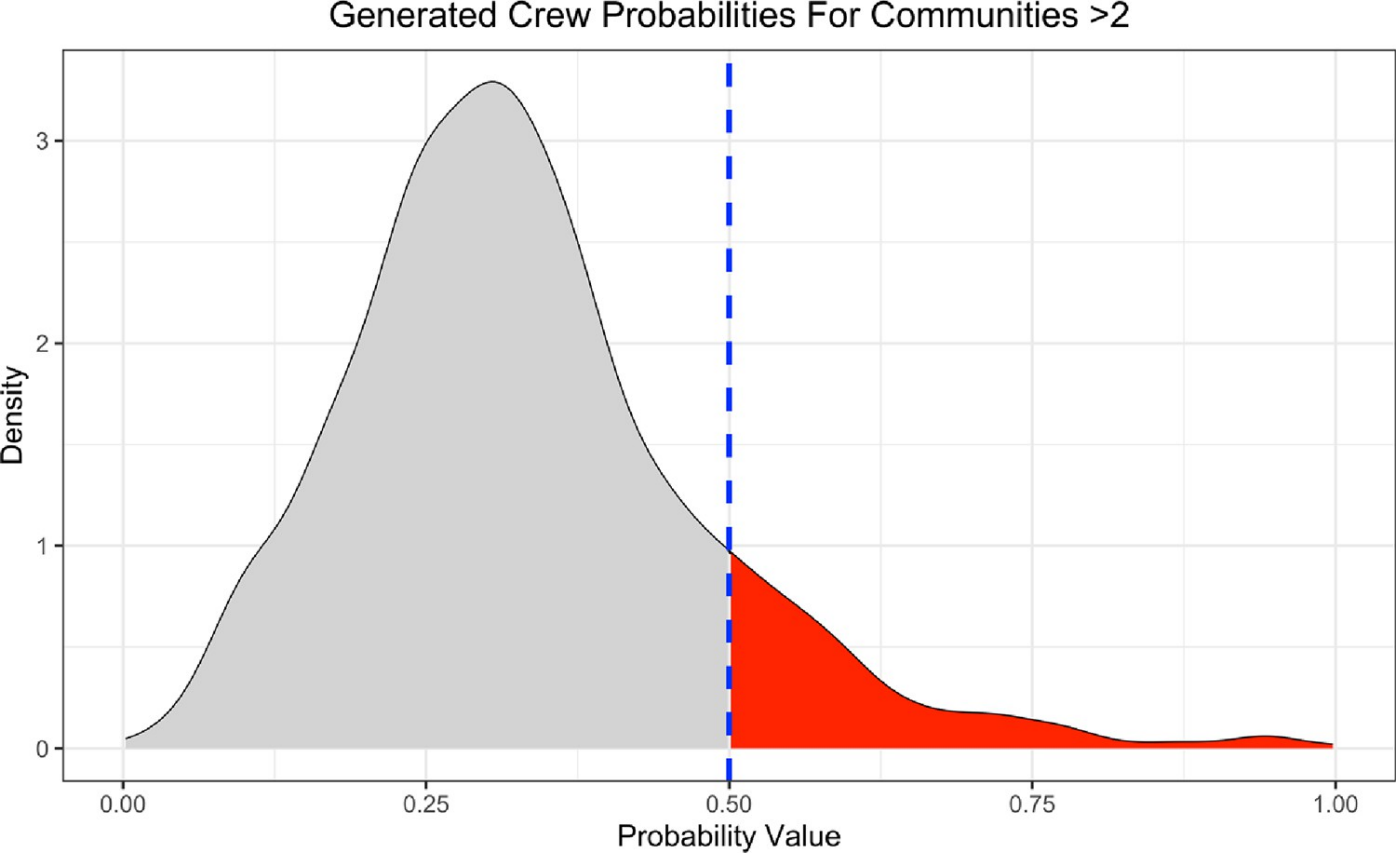
Distribution of crew probabilities for communities of size greater than two. The blue line indicates the cut-off value of 0.5 and the red region indicates that points that fall in that region were identified as crews.

Each of the 1,331 communities were provided with a crew probability. The overall distribution of probabilities is depicted in Fig 2. A cut-off point of 0.5 was chosen to represent communities who were likely crews.

There are 160 communities (a total of 1,156 officers) with a crew probability of greater than or equal to 0.5. Of the 29 officers in known crews in the case studies, 15 were identified as crew members by this analysis including 12 of 17 Watts crew members, and 3 of 5 Skullcap crew members; one of the Watts crew members was not part of the co-assusal network due to a lack of meaningful edges. Detected communities include those that contained the majority of the Skullcap crew and the Watts crew. No Austin-Seven officers were detected by this analysis.

#### Descriptive crew properties and activity

The 1,156 officers in crews comprise approximately 3.43% of all CPD officers in the dataset. The majority of officers in crews (53.8%) are white men with, on average, 21 years on the job. As compared to officers more generally, officers found in crews were more likely to be Hispanic and less-likely to be white. Approximately 20% of crew officers were Black. Additionally, officers in crews are on average 9 years younger than their non-crew peers.

**Fig 3:**
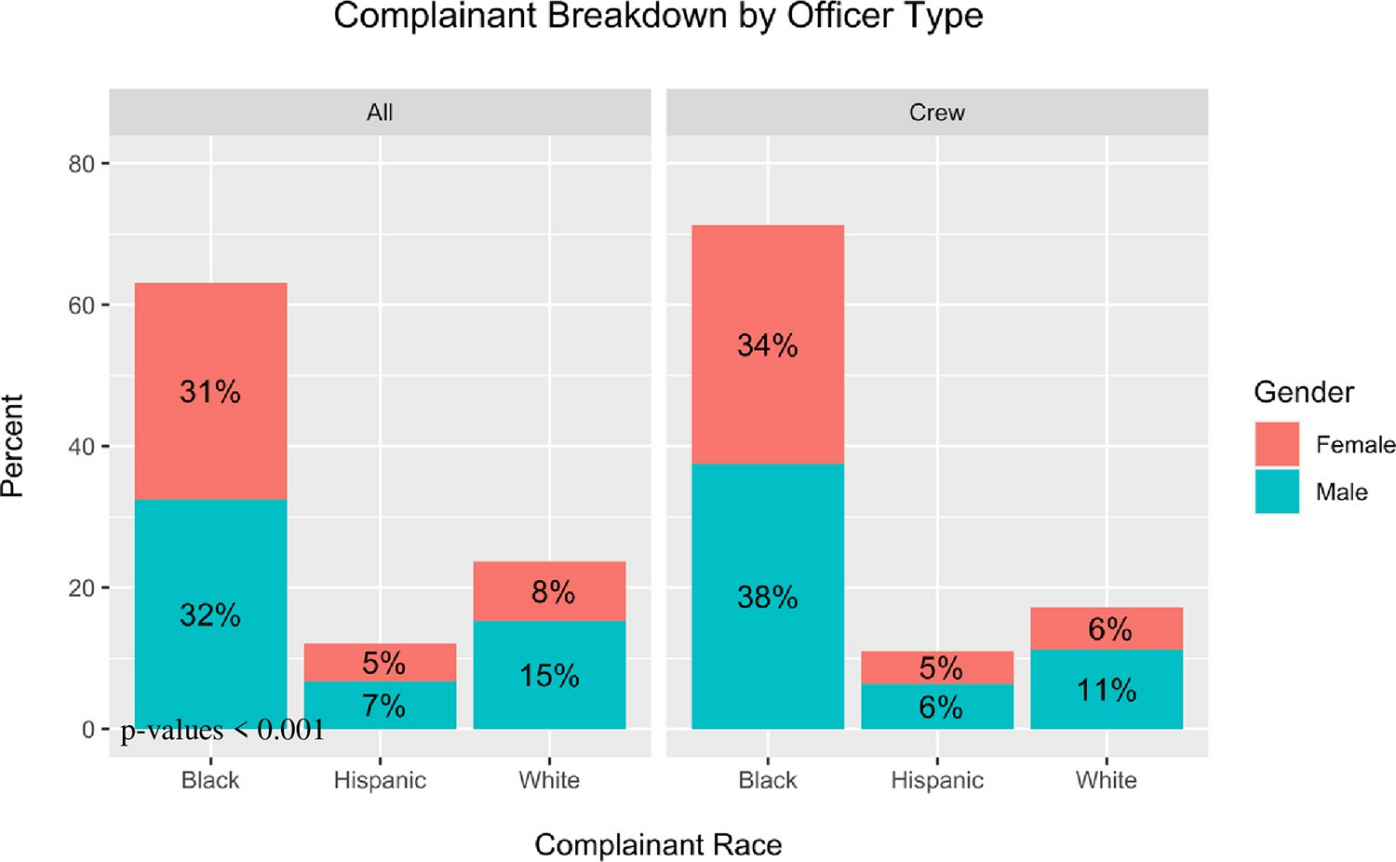
Complainant breakdown for all officers and for officers in crews. Differences of means between each race and gender combination is statistically significant at a 0.1% level, with the exclusion of the difference in means of Hispanic females which is statistically significant at a 5% level, and the difference in means of Hispanic males which is statistically insignificant.

*Alleged misconduct activity*. The detected crews were mentioned in a disproportionate number of complaints against CPD officers during the observation period. Officers in crews were listed on 14.7% of all complaints and 23.8% of all use of force complaints. The accused misconduct associated with crews also seems to be directed more towards Black as compared to Hispanic or white civilians. For cases where complainant information (race and gender) are known, officers in crews are tied to 17.4% of complaints filed by Black males and 16.6% of complaints filed by Black females. They are also responsible for 14.2% of complaints filed by Hispanic males, 13.0% of complaints filed by Hispanic females, 11.0% of complaints filed by white males, and 10.8% of complaints filed by white females. A comparison of the distribution by crew complaints with the overall complainant distribution shows a noticeable rise in the complaints filed by Black individuals, and a noticeable decline in complaints filed by White individuals (Fig 3).

Officers in crews were also listed as parties on 27.3% of city awards and settlements in civil settlements between 1993 and 2016. With respect to city payouts on a per officer basis, the average settlement for an officer in a crew was more than four times as high as their non-crew peers: officers in crews saw an average settlement of $112,343 whereas the average payout for an officer not in a crew was only $26,425 (p-value < 0.001). Because high misconduct activity was incorporated into the model, showing that these officers are responsible for a large portion of misconduct does not verify any core assumptions that officers in crews are more active. Rather, it further illustrates the disproportionate and negative impact that officers and crews had on the city.

*Arrest activity*. Between 2001 and 2018, a total of 17,558 unique CPD officers made at least one arrest; officers in crews are listed on 15.2% of all the arrests in the dataset. Expressed as a ratio, officers in the 160 crews had an arrest rate more than three-times higher than non-crew officers. One may argue that the similar fraction of misconduct allegations and arrests may indicate that officers who are dispatched to higher crime areas that lead to higher levels of activity; however, increased suspect interaction and a high number of arrests could also be indicative of crew behavior as well. In the case of at least one crew—The Watts Crew—there have been more than 100 exonerations to date suggesting that these arrest patterns should be more closely examined to determine if they are, in fact, false arrests.

*Tactical response reports*. The majority of officers in the detected crews—784 of the 1,156 officers, roughly 67 percent—filed at least one TRR between 2004 and 2016. All together, these officers in crews filed 10.4% of all TRRs during this time, including 12.5% of all firearm discharges, and 9.7% of all taser discharges. This translates to crew officer involvement in 20.2% of all firearm discharge incidents. Furthermore, of the 753 TRRs that reported a subject was shot by the police, 92 were filed by officers in crews. This translates to crew officer involvement in 72 of 318 shooting incidents (22.6%). While TRR data was not directly incorporated into the model, there is likely a strong correlation between the number of received TRRs and complaints.

#### Example crews

Even though our statistical approach was guided by validated cases of crews in Chicago, our results do not necessarily mean that all 160 crews are, in fact, similar to the Skull Cap, Watts, or Austin Seven crews. There might be qualitative differences between these validated cases and the crews detected using our statistical approach. Even though we have erred on the side of conservative estimates, it is possible that the communities we detected are an artifact of other organizational factors such as unit assignment.

We see the community detection approach advanced here as a first step in identifying potential crews which lend itself to further research, investigation, and validation. A natural point of departure is to zoom into communities to better understand how their detection does or does not align with the sorts of police misconduct and behaviors that might warrant them being signaled as a crew. In the remainder of this section, we take a more detailed look at three crews selected randomly from different areas of the probability spectrum described above (one high, one intermediate, and one low). Given these communities have not been formally investigated nor have complaints necessarily been sustained, we have anonymized specific details of events and officers.

### Example 1: Community 424 (C424)—Crew probability of 95.094%

C424 consists of five officers and appears to have been most active between 1997 and 2002 in one police district. In total, these five officers have received 281 total allegations during 214 separate incidents. There are 58 complaints that list more than one member of this community. Of these 58 complaints that happened within the crew, 27 were for use of force and 10 were for illegal search. While 40 of the complaints were not sustained, one complaint involving multiple crew members which alleged criminal misconduct was sustained. Officers of C424 are listed on four city settlements, for a total of $142,500. Officers in this crew have appeared in the media several times in connection with these allegations; one officer of this crew later faced a conviction relating to illegal activities while still an officer.

### Example 2: Community 15 (C15)—Crew probability of 73.212%

C15 consists of 12 officers and appears to have been most active between 2011 and 2013 in a single police District. In total, these 12 officers have received 269 total allegations during 170 separate incidents. There are 61 complaints that list more than one member of this community on same complaint. Of these 61 complaints, 16 were for use of force and 23 were for illegal search. Fourteen of these complaints within the crew were not sustained, yet 37 were dismissed because the complainant never signed an affidavit. Nearly all the complaints with complete information (53 of the 54 complaints) were filed by Black complainants, including 34 by Black men. Officers of C15 are listed on eight city settlements, for a total of $1,716,130. In two documented instances, members of this crew were caught telling other officers to turn off their body cameras during police raids or other interactions with civilians.

### Example 3: Community 413 (C413)—Crew probability of 53.707%

C413 consists of 6 officers who were most active between 2001 and 2006 in and around a public housing project. Many former residents of the housing projects made allegations of officers subjecting Black males to strip searches and have led to a lawsuit filed by tenants for improper or false arrests. Six of the officers in this crew have received 185 total allegations during 136 separate incidents. There are 25 complaints that list more than one member of this community on the same incident. Of these 25 within crew complaints, 8 were for use of force and 11 were for illegal search. 18 of the complaints were not sustained and no internal complaint was sustained. Officers of C413 are listed on three city settlements, for a total of $26,125. Much like the above communities, officers in C413, specifically one officer, have appeared in several high-profile complaints cases. In one instance, several officers in C413 were named on a complaint with multiple allegations including the illegal detention and abuse of an individual illegally taken from their home; this instance included threatening the civilian with a dangerous non-police issued weapon. In an instance a few years later, another officer in C413 was sued for the abuse of a protester.

## Conclusion

Policy debate surrounding police violence and misconduct often centers on “problem officers,” the proverbial “bad apples,” whose levels and types of misconduct result from some error in judgment, character flaw, predisposition, bias, or lack of self-control. Yet, police departments across the country have also experienced instances in which groups of officers coalesce into cliques or small groups with the explicit intention of engaging in misconduct, including outright criminal behavior, and the concealment of such activity. The extent of such “crews,” as we refer to them, is largely unknown, often only coming to light by the way of whistleblowers or investigative journalists. Using a unique set of data on the Chicago Police Department and known cases of crews, this study employed community detection algorithms to determine the existence of possible crews and the extent of their misconduct behavior. The methods advanced here identified 160 officer crews in CPD, consisting of 1,156 officers. Despite comprising less than 4% of all Chicago police officers, these crews were involved in oversized amounts of police violence and misconduct. Our estimates suggest that these crews were responsible for approximately a quarter of all use of force complaints, city payouts, and police shootings, as well as a disproportionate amount of the complaints generated by and arrests of Black and Hispanic civilians.

Our approach and the methods are intended as a way to detect the existence of possible crews within a large police agency. While the present data do not permit us to predict future crews, identify causal links, or unpack the conditions under which such crews form or evolve, our results demonstrate that identified crews generate massive disparities in criminal justice outcomes and police misconduct. Importantly, our approach is not intended to (nor should it) assess the actual culpability of officers involved in reported instances of wrongdoing. Thus, while our analyses are intended as a jumping off point for the identification of possible police misconduct, actual legal investigation and inspection would be required to determine if such groupings constitute crews such as those that have previously come to light.

Our study is not without limitations. First and foremost, police misconduct is underreported and, as known cases have demonstrated, crews often go to great lengths to conceal their behaviors. The data we use are derived from complaints filed by civilians which, while they report on behavior deemed problematic, might not always capture actual misconduct. While prior research suggests a correlation between complaints and actual misconduct, future research would do well to investigate additional cases of verified misconduct behaviors. Overall, this suggests that we likely underestimate actual misconduct.

Second, the lack of training data (i.e., known crews) presents a limitation on the generalizability of these findings. Our approach was based on three crews that were “caught,” and it is possible that these three crews are fundamentally different from others which is what led to their detection–i.e., they somehow represent “failed” crews. While steps were taken to address this concern, future research should consider expanding the sorts of crews or cases that help inform community detection. Another limitation from the lack of training data is the proposal of an equally weighted index function. Calculating non-equal weights or perhaps even a non-linear specification of the function could be a valuable source of future research.

Third, our study’s focus on the detection of crews confirms their presence but does not answer questions pertaining to the emergence or evolution of such crews over time. In this sense, our study is an important exploratory step. Future research might consider adding additional data to those examined here to unpack the dynamics of these networks over time. Such an analysis might also open up unique historical moments which impacted policing behaviors and practices. Additionally, as future known crews are uncovered, advanced temporal analysis, such as partitioning data into separate time periods for separate analysis, may become feasible.

Finally, our analyses serves as a jumping off point with respect to the types of observed behaviors (those reported in the form of complaints) highlighting the need for targeted further investigation. Specific areas of investigation may be needed into the types of alleged misconduct, the linking of these crews to other problematic police behaviors (e.g., sexual assault, theft, drug trafficking, etc.), the role of “adjacent” officers, the formation of such crews, as well as the variance of behavior by policing assignments, geography, and contexts (e.g., gang units, patrol, assignment to public housing, etc.). Unfortunately, any attempt at advanced classification is also hindered by data constraints regarding the limited information of each complaint. For example, it is almost a certainty that not every use of force complaint is of the same severity, and without more detailed information regarding the content of the allegations, performing type classification would be ineffective.

The impact of identifying crews in the Chicago Police Department, like the one led by Ronald Watts for over a decade, extends far beyond the numbers. Past victims and future victims, as well as entire communities and departments are all impacted by the effects of such severe acts of police misconduct. While identifying these crews is certainly not a solution for police misconduct, it can certainly be an impactful place to start identifying key sources of harm. Nearly all the instances of crews cited here, not to mention similar cases in Los Angeles, Oakland, New York, and other cities across this country—were brought to light only through whistle blowers, missteps by rogue officers, journalists, or victims willing to come forward. The analysis presented here demonstrates the possibility of systematically using data to identify networks within a police department that may prove, when investigated, to be criminal crews. However, even the best efforts at identifying and validating such crews will only go so far without the capacity to fully investigate such cases and, when deemed necessary through due process, discipline, dismiss, or otherwise hold accountable the officers involved.

## Supporting information

S1 File (DOCX)
