# Correction: Identifying misconduct-committing officer crews in the Chicago police department

**DOI:** 10.1371/journal.pone.0283765

**Published:** 2023-03-23

**Authors:** The PLOS ONE Editors

This article’s [1] Competing Interests statement is updated to:

RS is affiliated with the Invisible Institute, for which he was a co-founder. This does not impact the author’s adherence to PLOS policies.

Before this article was published, the *PLOS ONE* Editors identified reporting issues for which updates were needed to bring the article into full compliance with the article’s standards and requirements. Unfortunately, the article was published in error before the *PLOS ONE* Editors had discussed these issues with the authors. We apologize to the authors and to readers for this error. *PLOS ONE* followed up to discuss the pending issues with the authors after the article was published, and we highlight the following points here in order to clarify and correct aspects of the study’s reporting.

- In this article, the terms ‘crew’ and ‘misconduct crew’ refer specifically to cohorts of police officers linked via misconduct allegations. The article does not clarify whether professional affiliates (e.g., partners, team members, officers working on joint assignments) of individuals named in misconduct allegations were considered as crew members if they themselves were not accused of misconduct. Also, for crews identified in the article, it is unclear from the reported data whether there was substantiated evidence of coordinated group-level activity vs. individual-level involvement by multiple officers.
- Except for instances where the article specifies misconduct was confirmed via a court ruling or conviction, the term ‘misconduct’ in this article [1] should be interpreted as referring to allegations of misconduct rather than confirmed misconduct. Declarative statements in [1] referring to police misconduct should instead be framed around potential misconduct or allegations of misconduct, e.g., “crews engaging in misconduct” should instead be read as, “crews linked via misconduct allegations”.
- As is clearly stated in the second paragraph of the Conclusion section [1], the study design did not allow for investigation of causal relationships. As such, statements interpreting the study’s findings should be read as referring to correlative relationships.
- Since many data in the study pertain to allegations rather than confirmed, convicted misconduct cases, the title and conclusions overstate the results. The conclusions should refer to ‘existence of possible crews and the extent of their **alleged** misconduct behavior’. This is discussed on pages 8 and 17 of the paper, but the editors would have encouraged additional language in the introduction or framing of the paper.
- The article discusses the possibility that misconduct is likely underestimated, but this was not balanced by discussion of the possibility that data based on complaints and allegations may result in overestimates if misconduct claims are not substantiated or confirmed. Furthermore, it is unclear from the article whether, based on available data, one can distinguish between group involvement in misconduct vs. individual-level misconduct for which groups are named in allegations because partnerships and group assignments had multiple individuals on site together at the time of the incident. This issue was not raised by reviewers or editorial board before publication and would require additional data and analyses, but some might consider this a limitation of the study.

In addition, several statements in the article did not cite source information, and References 17, 54–59 are incomplete in [1]. Readers should contact the corresponding author with any questions about missing reference details and should take caution in interpreting informative statements in the article that are not supported by cited sources or reported data.
