# Supplementary material for: Identifying misconduct-committing officer crews in the Chicago police department
Source: PLoS One. 2022 May 4;17(5):e0267217. doi: 10.1371/journal.pone.0267217 (PMC9067648; doi:10.1371/journal.pone.0267217)
Supplement: S1 File — (DOCX) [file pone.0267217.s001.docx]

**Appendices and Supplemental Information**

Identifying Misconduct-Committing Officer Crews in the Chicago Police Department

Akshay Jain^1^, Rajiv Sinclair^2,3^, Andrew V Papachristos^1,4,5*^

^1^ Northwestern Neighborhood & Network Initiative,

^2^ The Invisible Institute

^3^ Public Data Works

^4^ Northwestern University, Department of Sociology

^5^ Northwestern University, Institute for Policy Research

^*^ Corresponding author.

# Appendix A

The following is a degree-distribution density curve for the network of 11,226 officers. The dashed-blue line denotes the mean degree, which is approximately 4.69. Meanwhile, the mode degree is 1 and the median degree is 2. The minimum degree is 1 and the maximum degree is 60.

**Table A-1. Unweighted Degree Distribution of Complaints**


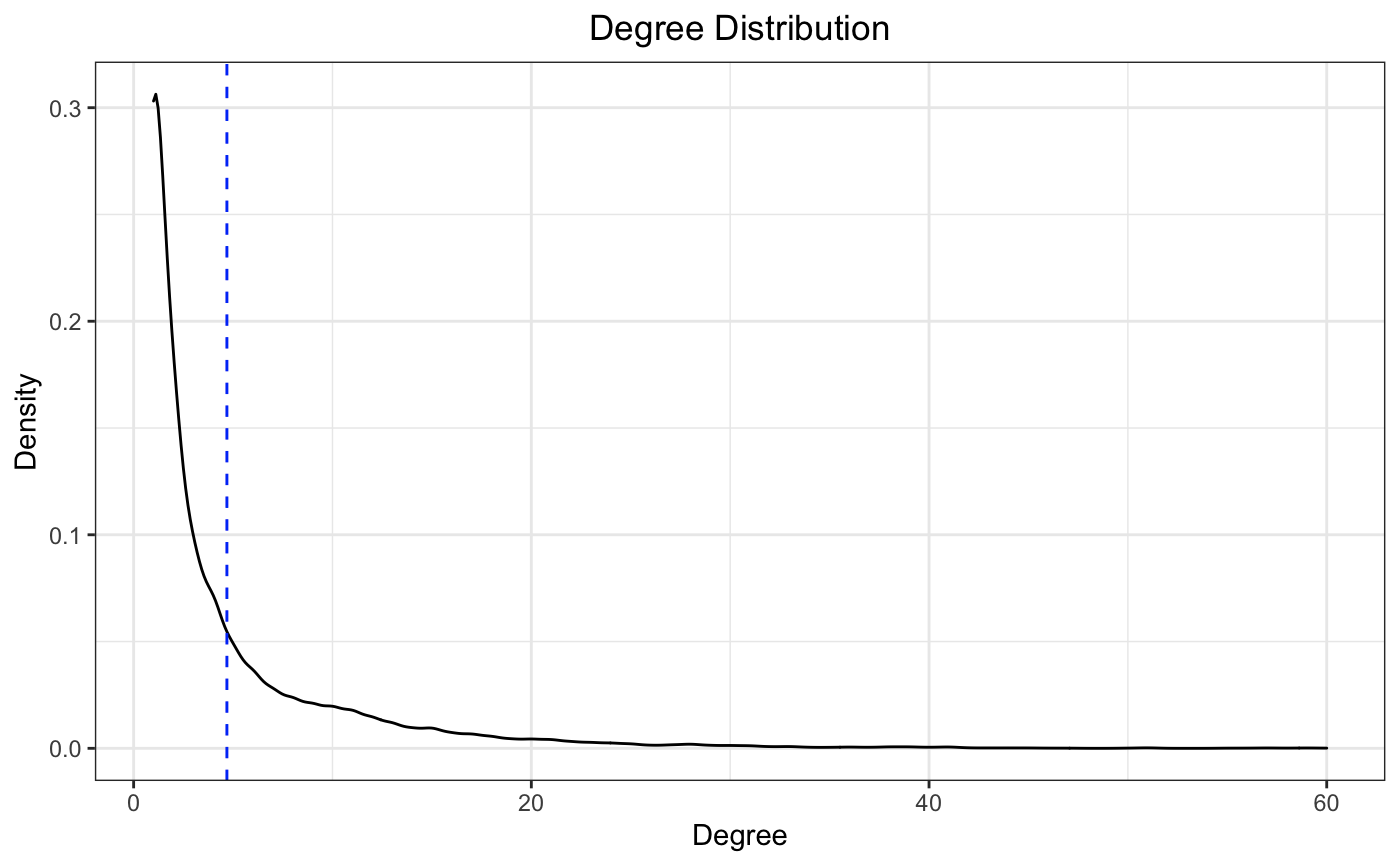


The following is a weighted-degree-distribution density curve for the same network. The dashed-blue line denotes the mean weighted-degree, which is approximately 19.30. The median weighted-degree is 8.33. The minimum weighted-degree is 2.02 and the maximum weighted-degree is 568.19.

**Table A-2. Weighted Degree Distribution of Complaints**

**
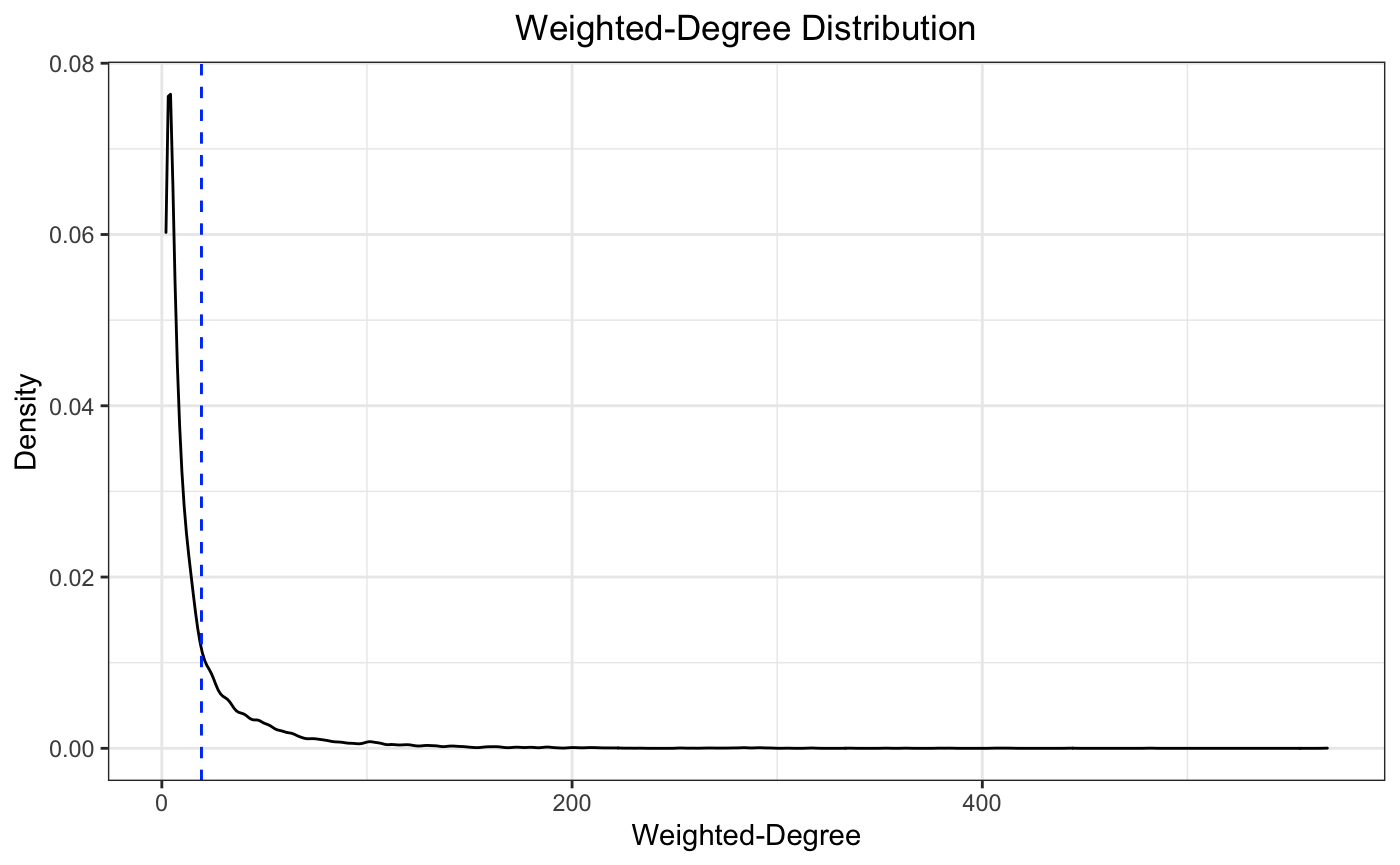
**

# Appendix B

*Louvain Method*

The Louvain method, created in 2008, was designed to identify community structures in large networks.[1][2] Louvain community detection functions as a greedy optimization method. In other words, it seeks to maximize the modularity of each detected community through an iterative algorithm that allocates nodes to communities until there is no positive change in modularity. Modularity is a metric designed to measure the division of graphs into different tightly knit modules, or communities.[3] Graphs with a high modularity score will have dense connections within each module and weak connections (if any) between modules. This method automatically calculates the ideal number of communities, which is perfect for our purposes as we do not know how many communities exist. The Louvain method excels at finding communities at a computationally faster rate than other algorithms, specifically in large networks with weighted edges. As such, it is our a priori prediction that the Louvain method will perform best.

*K-Clique Percolation*

A clique within a graph is defined by a fully connected subgraph, where each node in the sub-graph is connected to every other node. A k-clique community is the union of all cliques of size k that can be reached through adjacent (sharing k-1 nodes) k-cliques. In this paper we use a K-size of 3, meaning we will only obtain cliques of size 3 or greater. One main problem with this method, in addition to the computational expense and complexity, is the lack of representation of crews. While it is conceivable that some crews are indeed represented as cliques, most are probably not. Not even the Watts crew, the prime example for this paper, is represented by a clique. As such, it’s likely that this method will not perform as well as the others.

*Label Propagation*

Label propagation is designed to provide each node with the label possessed by most of its neighbors. Tightly knit communities will result in a quicker convergence to a common label among all members. This method typically is an iterative algorithm that can be either synchronously or asynchronously; however, due to the benefits of each type, we use a semi-synchronous approach that combines both types; however, it does not consider edge weights.[4] Similarly to the Louvain method, it does not require any a priori information regarding the number of communities; however, one major drawback is the inability to find one true solution, as the algorithm instead produces a combination of many solutions.[3]

*Clauset-Newman-Moore Greedy Modularity Maximization*

Similar to the Louvain method, the Clauset-Newman-Moore method is also a greedy optimization algorithm with respect to modularity. It's different from Louvain in that it is a hierarchical algorithm that joins pairs of communities to increase modularity.[5] This algorithm does not consider edge weights and was the most computationally expensive of all the selected methods. We expect it to perform similarly to the Louvain method; however due to the lack of any hierarchical organization in the CPD network, it's unlikely that the CNM method will outperform the Louvain method.

*Community Detection Evaluation*

To measure the success of each algorithm, we compare the generated communities to three existing communities of varying sizes that we know exist: the Watts crew (seventeen total officers), the "Austin-Seven" (seven total officers) and the "Skullcap Crew" (five total officers).

Each model's performance will be evaluated using Jaccard index which will reward proper classifications and penalize false classifications. The Jaccard index is designed to measure the similarity between two sets, A and B:

J = $\frac{|A\cap B|}{|A\cup B|}$

Our goal is to identify the model that produces the largest Jaccard indices for each of the three known communities. We will choose the predicted community that contains the highest number of officers who are actually in the community.

The following table is the Jaccard Index scores for each crew and each method. It shows that the Louvain method at a resolution of 0.0075 exceedingly outperforms the other methods for each crew. The resolution of 0.0075 was chosen arbitrarily for the sole purpose of evaluating model performance.

**Table B-1. Community Detection Method Test**

| **Community Detection Method Test (Jaccard Index)** | | | | |
| --- | --- | --- | --- | --- |
| **Crew** | **Louvain** | **K-Clique** | **Label Propagation** | **CNM** |
| Watts | 0.55 | 0.53 | 0.58 | 0.30 |
| Austin-Seven | 0.36 | 0.002 | 0.20 | 0.04 |
| SkullCap | 0.50 | 0.002 | 0.12 | 0.01 |
| Average | 0.47 | 0.18 | 0.30 | 0.12 |

At this point, it is clear that the Louvain method is the best option; however, we have to ensure that we select the best resolution. To do so, we will perform a similar Jaccard Index test on Louvain algorithms of differing resolutions. Smaller resolutions indicate an increased ability to detect smaller communities. Below are the results from the Louvain resolution test.

**Table B-2. Louvain Resolution Test**

| **Louvain Resolution Test (Jaccard Index)** | | | | | | |
| --- | --- | --- | --- | --- | --- | --- |
| **Crew** | **0.05** | **0.02** | **0.01** | **0.0075** | **0.005** | **0.0025** |
| Watts | 0.52 | 0.50 | 0.52 | 0.55 | 0.58 | 0.26 |
| Austin-Seven | 0.12 | 0.16 | 0.36 | 0.36 | 0.29 | 0.36 |
| SkullCap | 0.09 | 0.14 | 0.36 | 0.50 | 0.60 | 0.40 |
| Average | 0.24 | 0.27 | 0.41 | 0.47 | 0.49 | 0.34 |

| **Louvain Resolution Test (Jaccard Index)** | | | | | | |
| --- | --- | --- | --- | --- | --- | --- |
| **Crew** | **0.0075** | **0.007** | **0.0065** | **0.006** | **0.0055** | **0.005** |
| Watts | 0.55 | 0.52 | 0.58 | 0.60 | 0.52 | 0.58 |
| Austin-Seven | 0.36 | 0.36 | 0.36 | 0.36 | 0.36 | 0.29 |
| SkullCap | 0.50 | 0.50 | 0.50 | 0.50 | 0.50 | 0.60 |
| Average | 0.47 | 0.46 | 0.48 | 0.49 | 0.46 | 0.49 |

From the above table and previous literature, it's clear that lower resolutions perform better at identifying smaller communities, but worse at identifying larger communities. As such, we will move forward with the Louvain resolution of 0.006.^^[[1]](#footnote-1)^^

# Appendix C

**Table C-1. Determination of K-Value using average silhouette analysis**

**
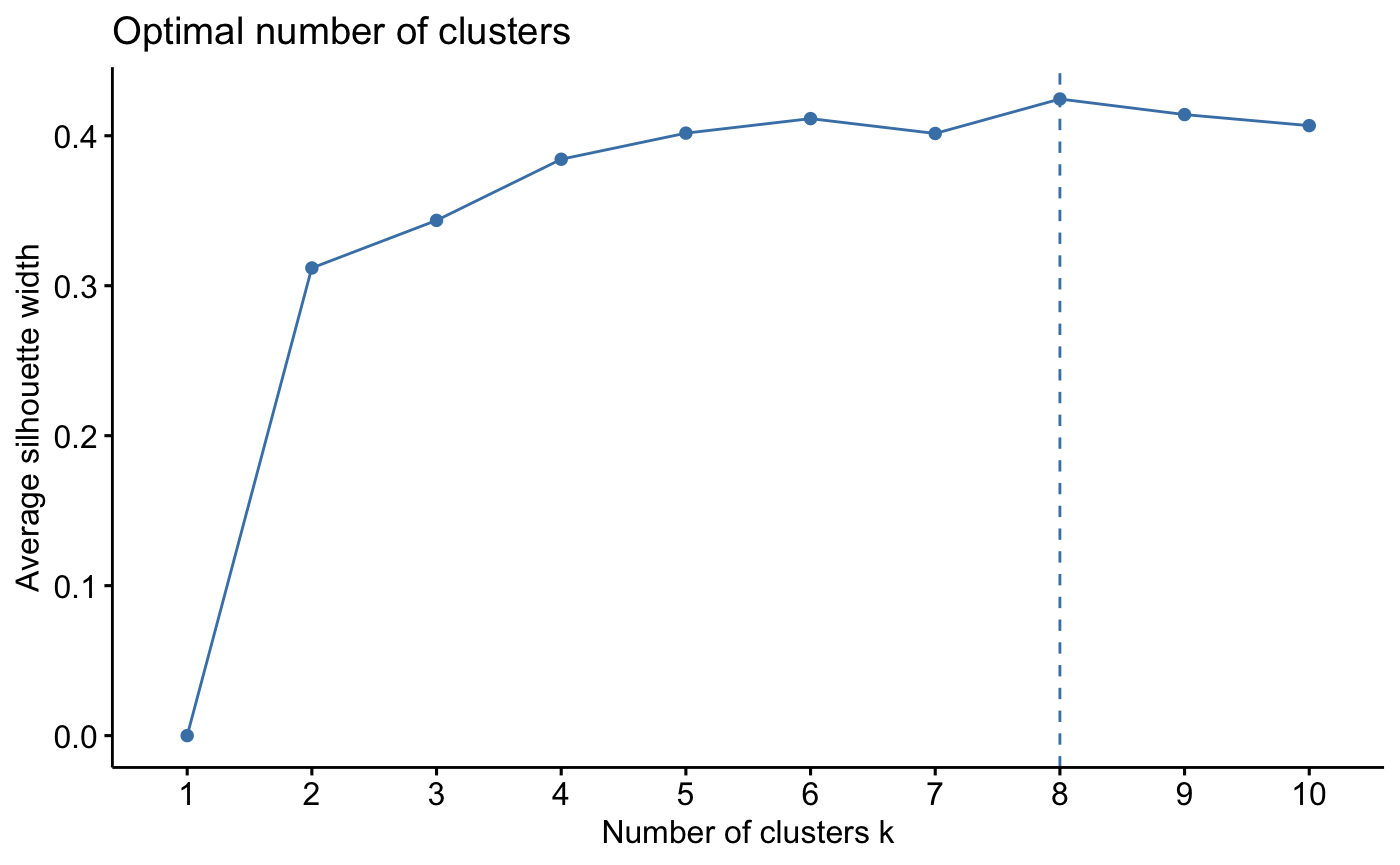
**

The silhouette curve indicates that eight clusters maximize the average silhouette score.

# Appendix D

**Table D-1. The values in the below table represent the average results of all officers in each category.**

| Officer Category | Number of Allegations | Percent of Complaints Committed Alone | Percent of Complaints Sustained | Percent of Complaints Unfounded / Exonerated | Percent Type 1 Complaints | Percent Type 2 Complaints | Percent Type 3 Complaints | Percent Type 4 Complaints | Percent Type 5 Complaints |
| --- | --- | --- | --- | --- | --- | --- | --- | --- | --- |
| 1 | 3.56 | 74.93 | 45.21 | 17.08 | 9.66 | 0.18 | 0.84 | 17.16 | 71.53 |
| 2 | 1.37 | 26.02 | 46.34 | 17.07 | 5.69 | 82.93 | 0.41 | 10.16 | 0.81 |
| 3 | 4.29 | 57.28 | 69.00 | 9.08 | 24.41 | 0.38 | 1.10 | 68.02 | 5.59 |
| 4 | 8.41 | 19.08 | 4.53 | 29.56 | 64.18 | 0.49 | 2.66 | 28.37 | 3.59 |
| 5 | 0.01 | 0.10 | 0 | 0.01 | 0.07 | 0.02 | 0 | 0.11 | 0.01 |
| 6 | 31.93 | 31.27 | 7.05 | 26.94 | 50.08 | 0.46 | 6.43 | 35.83 | 6.62 |
| 7 | 5.13 | 45.17 | 9.14 | 25.83 | 19.66 | 0.43 | 42.60 | 32.70 | 4.26 |
| 8 | 6.56 | 29.25 | 7.83 | 33.80 | 15.73 | 0.33 | 2.61 | 76.09 | 4.64 |

**Works Cited**

1. Campigotto R, Céspedes PC, Guillaume J-L. A generalized and adaptive method for community detection. ArXiv Prepr ArXiv14062518. 2014.

2. De Meo P, Ferrara E, Fiumara G, Provetti A. Generalized louvain method for community detection in large networks. 2011 11th international conference on intelligent systems design and applications. IEEE; 2011. pp. 88–93.

3. Newman ME. Modularity and community structure in networks. Proc Natl Acad Sci. 2006;103: 8577–8582.

4. Cordasco G, Gargano L. Community Detection via Semi-Synchronous Label Propagation Algorithms. ArXiv11034550 Phys. 2011 [cited 9 Apr 2022]. doi:10.1504/..045103

5. Clauset A, Newman MEJ, Moore C. Finding community structure in very large networks. Phys Rev E. 2004;70: 066111. doi:10.1103/PhysRevE.70.066111

1. Expansion to further decimal points prior to rounding indicates better performance of resolution 0.006 than of resolution 0.005. [↑](#footnote-ref-1)
